# Apparent differences between human and chimp proteomes are reduced when considering human population: Human specific variants are enriched in disordered and compositionally biased regions

**DOI:** 10.1371/journal.pone.0328504

**Published:** 2025-07-31

**Authors:** Pablo Mier, Miguel A. Andrade-Navarro, Enrique Morett

**Affiliations:** 1 Andalusian Centre for Developmental Biology (CABD, UPO-CSIC-JA), Faculty of Experimental Sciences (Genetics Area), Universidad Pablo de Olavide, Seville, Spain; 2 Institute of Organismic and Molecular Evolution, Faculty of Biology, Johannes Gutenberg University Mainz. Hans-Dieter-Hüsch-Weg 15, Mainz, Germany; 3 Departamento de Ingeniería Celular y Biocatálisis, Instituto de Biotecnología, Universidad Nacional Autónoma de México (UNAM), Av. Universidad, Cuernavaca, Morelos, México; UMR-S1134, INSERM, Université Paris Diderot, INTS, FRANCE

## Abstract

Humans exhibit significant differences from other primates in anatomy, physiology, behavior, and culture, despite having similar genomes. Understanding the genetic basis of these unique human traits has long been a goal of science and philosophy. Previous studies, including the comparison of the reference genomes, showed a high degree of sequence identity between the proteomes of humans and chimpanzees (*Pan troglodytes)*, suggesting that differences may lie in gene regulation rather than protein function. To pinpoint human-specific protein mutations with possible relevance for human-specific traits, we went further in the study of human-chimp proteome differences by taking into account human genetic variation data from the Genome Aggregation Database (gnomAD) at protein-coding genes. We additionally included 11 primate genomes to identify human-specific amino acids. Results showed that human-specific positions were dramatically reduced when considering population diversity. Our analysis identified 6210 human-specific amino acid substitutions across 4475 proteins. Interestingly, these residues are enriched on disordered and compositionally biased regions, suggesting a role in protein regulation instead of a catalytic or structural one. Accordingly, the set of proteins holding them was significantly enriched in proteins with disordered regions and with protein binding functions. We found that a subset of these residues is not only different in humans but also conserved across non-human primates, further supporting their potential importance in making us different from other primates.

## Introduction

Humans are dramatically different from other primates in terms of anatomy, physiology, nutrition (we are the only species that universally cook our food), reproduction, language, and behavior [1]. Among the particularities of our species are the generation of culture, art and science, taking care of our kind, and a long etcetera. To understand the critical characteristics that make us humans has been a long-time goal not only of science but also of philosophy and theology [2]. These differences are not easily explained by our genomes, which are very similar to those of other primates and have varied accordingly to the expected rate of evolutionary divergence [3].

Our closest living relatives are the two species of chimpanzees from which we diverged no more than 8 million years ago [4]. The first molecular studies of the divergence in protein sequences showed a high degree of similarity between humans and chimpanzees [5], which was accurately assessed with the completion of the sequencing of both genomes [4,6]. Now it is well documented that there is around 99% identity of the nucleotides that can be unambiguously aligned between human and chimpanzee. Regarding the one-to-one protein orthologous sequences, an astonishing 29% are identical, while the rest have no more than 1% of amino acid substitutions when comparing the reference proteomes, and there is a very high probability that these differences occurred by random genetic drift which, in general, does not impact the phenotype; chimpanzees diverged about as much from the common human-chimpanzee ancestor as humans did [7].

The conclusion that emerged from these findings was that the genomic variation that accounts for the species differences mentioned above must be mainly in regions other than protein-coding sequences. Accordingly, accurate *de novo* long read sequencing of primate genomes has unambiguously shown that there is a high degree of structural variation, as deletions, inversions, translocations, chromosome rearrangements, and distribution of repetitive sequences [8]. Therefore, by far the greatest differences in the genomes of primates occurred in the non-coding regions, some of which control gene expression. This was predicted in 1975 in a seminal paper by King and Wilson [5]; they suggested that “evolutionary changes in anatomy and way of life are more often based on changes in the mechanisms controlling the expression of genes than on sequence changes in proteins”. Therefore, they proposed that “regulatory mutations account for the major biological differences between humans and chimpanzees”.

However, the question remains as to whether relevant regulatory mutations between humans and chimpanzees can be found at the proteome level. In this work, we propose that recent advances in resources providing information on population variation facilitate this task.

In recent years there has been a large number of projects centered in the analysis of the human population genomic diversity. The 1000 genomes project was the first large effort to characterize human genetic variation by whole genome sequencing of unrelated individuals of different geographic regions [9] and several other large projects, both by whole genome sequencing and by exome sequencing have informed us of the great wealth of genomic diversity in the human population [10].

The aim of this work was to make a new, more stringent, evaluation of human-specific amino acid positions taking into account the variability observed in the human population. Our goal was to rule out as human-specific amino acids at positions that, being different at the level of the reference genome, are polymorphic, with some individuals presenting variants found in chimpanzees (*Pan troglodytes*) or other primates. The rationale was that the early assessment of the genetic differences between human and chimp was limited to comparing their respective reference genomes, but now we have a very large number of genomes from populations around the world. It is very likely that many of the differences found only with the human reference genome no longer hold when taking into account the large population variability. Similarly, the differences observed with the chimpanzee could be due to random drift of both species and many differences could be shared between humans and other primates, thus they are not truly human-specific. Using this approach, we reduced the number of human-specific substitutions by 95%, compared to the number of human-chimp substitutions obtained by comparing their reference proteomes. We hypothesized that this would allow us to make more relevant observations regarding the functional importance of these positions. Interestingly, the variants are enriched in disordered and compositionally biased regions, suggesting that they tend to be related to regulatory and interaction functions and less to catalytic and structural protein functions.

## Materials and methods

We downloaded the protein-coding sets of all 12 primates available in Ensembl genes 110 [11]: *Homo sapiens* (hsa, assembly GRCh38.p14), *Pan troglodytes* (ptr, assembly Pan_tro_3.0), *Gorilla gorilla* (ggo, assembly gorGor4), *Pongo abelii* (pab, assembly Susie_PABv2), *Pan paniscus* (ppa, assembly panpan1.1), *Chlorocebus sabaeus* (csa, assembly ChlSab1.1), *Nomascus leucogenys* (nle, assembly Nleu_3.0), *Macaca mulatta* (mmu, assembly Mmul_10), *Otolemur garnettii* (oga, assembly OtoGar3), *Microcebus murinus* (mmur, assembly Mmur_3.0), *Callithrix jacchus* (cja, assembly mCalJac1.pat.X), and *Papio anubis* (pan, assembly Panubis1.0). We took human as the reference and downloaded also from Ensembl the set of one-to-one orthologs between each of these species and human. Last, we downloaded a set of chromosome variation sites obtained from 125,748 human exomes, and the sites from exome calling intervals from 15,708 whole human genomes, from the Genome Aggregation Database (gnomAD) v2.1.1 database [12]. We imposed no filter to the minimum number of alleles for a variant to be considered. We additionally downloaded the 730,947 exomes and 76,215 whole-genome sequences from genomAD v4.0 [13].

Protein alignments were done with MAFFT v7.490 [14]. Gene annotation enrichments were performed in the Database for Annotation, Visualization and Integrated Discovery (DAVID v2025_1) [15], and with PANTHER 19.0 [16]. Positional annotations were downloaded from UniProtKB v2023_05 [17].

We aligned the set of proteins of the human reference proteome with their orthologous proteins in the reference proteome of the chimpanzee (*Pan troglodytes*). We produced 17,026 alignments from the set of one-to-one orthologs provided by Ensembl, in which 79.7% of the amino acid positions match. This result is very far from what is known about the similarity of human and chimpanzee sequences [7,18]. We observed high length variability in a small portion of the orthologs, probably due to an incorrect protein annotation, making the aligned positions not comparable. To avoid this issue, we repeated the alignments but considering only the pairs of orthologs with similar length (one ortholog with at least 80% of the length of the other). With this strategy, we produced 16,711 alignments of one-to-one orthologs.

## Results

### Human-specific residues are rare in the human population

We produced 16,711 alignments of one-to-one human-chimp orthologs with 95.92% matching positions (9,639,349 residues; see Methods), a result comparable to the previously reported human-chimp similarity. The 409,972 mismatched positions are distributed as follows: 78,098 positions with an amino acid in the human sequence and not in the chimp (19.04%), 209,384 positions with an amino acid in the chimp sequence and not in the human (51.07%), and 122,490 positions with different amino acid in each species (29.88%). The latter are the ones in which we will focus our analysis (S1 Fig).

We then used the information in the gnomAD v2 database to select a stricter set of amino acid differences between humans and chimps, by considering the variability of the human population in the mismatched positions. For each mismatched position, we obtained the ensemble of amino acids reported by at least one allele in the human population and, if the chimp amino acid was found in this ensemble, then the position was not considered a mismatch. This reduced the number of mismatches with the chimp proteome to 100,639 mismatches.

To expand the study to a comparison between the human proteome and the primate proteomes we included all of the remaining primates in the Ensembl database, a total of 10 more species. Per mismatch in the current set, we compared the amino acid in the human protein with the amino acid in the orthologous proteins of all of the primates. This further reduced the number of human-specific residues to 33,807 mismatches, in 8704 different proteins. By applying the gnomAD v2 ensemble of human amino acids per mismatch to the amino acids from all primates, we slightly reduced the number of remaining mismatches to 31,906 mismatches from 8456 different proteins.

We noticed that mismatches accumulated at the protein termini (S2 Fig), very likely due to the incorrect alignment of the sequences at these regions. We filtered out the mismatches in the first and last decile of the human proteins using relative length. The set of remaining mismatches contains 20,038 human-specific residues from 7052 different proteins (S1 File).

To avoid mistakenly identifying human isoforms as mismatches, we selected those that were relatively isolated in the sequence as opposed to happening in blocks. For this, we rejected mismatches if more than four occurred in a window of ten amino acids. This resulted in a dataset of 10,210 human-specific substitutions in 6243 different proteins. As a last step, we used the latest version of the gnomAD database (v4) to filter them further, yielding a dataset of 6210 human-specific substitutions in 4475 different proteins (S2 File).

**Fig 1:**
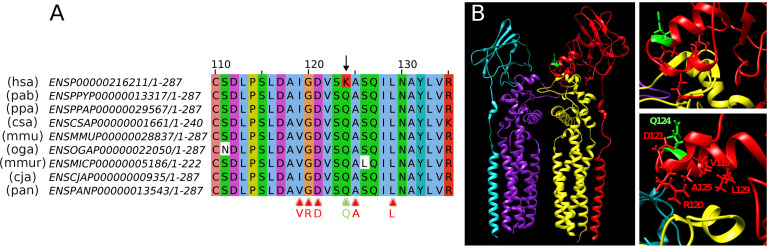
Example of an isolated human-specific mismatch in protein UPK3A. **(A)** Multiple sequence alignment of the UPK3A protein in primates; the arrow marks the human-specific mismatch in position 124. Equivalent positions in pig UPK3A, highlighted in panel B have been marked with triangles at the bottom of the alignment: none of them is different between human and all the other primates presented. **(B)** Uroplakin complex in pig: Uroplakin-2 in cyan; Uroplakin-1a in yellow; Tetraspanin in purple; Uroplakin-3a in red, with the position equivalent to human K124 (Q124) coloured in green. The side chains of Q124 and of residues close in sequence possibly involved in contacts with Uroplakin-1a are shown (the insets show the detail and a slightly rotated view). Residues and numbers refer to the pig sequence. The transmembrane helices of the complex are at the bottom of the structure.

We show an example of an isolated human-specific mismatch in human protein UPK3A (Uroplakin-3a; Ensembl:ENSP00000216211), K124, which is a Q in the non-human primates (Fig 1A). The amino acid change is probably given by a point mutation from glutamine (coded by CA(G/A), conserved in all non-human primates) to lysine (coded by AA(G/A), specific to the human protein). Uroplakin-3a forms part of the asymmetric unit membrane (AUM), a component of urothelial cells, which cover the urinary tract. The electron microscopy structure of pig’s Uroplakin-3a (86% identical to the human sequence; no experimentally-obtained structure available for the human protein) is available as part of the pig’s uroplakin complex in the AUM (PDB:8JJ5) [19], which includes Uroplakin-2, Uroplakin-1a and Tetraspanin. Pig’s Uroplakin-3a, like non-human primates, has a Q at position 124. In this uroplakin complex structure, Q124 is at the end of an arm formed by two helices (Fig 1B). Considering C-alpha-C-alpha distances, a number of residues surrounding this position in Uroplakin-3a are at a distance of less than 5A from residues in Uroplakin-1a (V119, R120, D121, A125 and L129; highlighted in Fig 1B). The human-specific mutation could be an adaptation to modify the interaction of Uroplakin-3a with Uroplakin-1a. The region could be functionally important since human UPK3A mutations I119T, G120E and D121Y are currently associated with Renal hypodysplasia/aplasia 1 (according to ClinVar) [20]. We used PolyPhen2 [21] to predict the effect of a mutation of K124 to Q in human Uroplakin 3A. The mutation is predicted to be benign suggesting that it has no structural effect. This was expected, considering that the other primates have this mutation. Therefore, we would not expect this mutation to have a strong effect on the primary function of the complex but rather on the dynamics of its formation.

### Human-specific residues are enriched in disordered and compositionally biased regions

**Fig 2:**
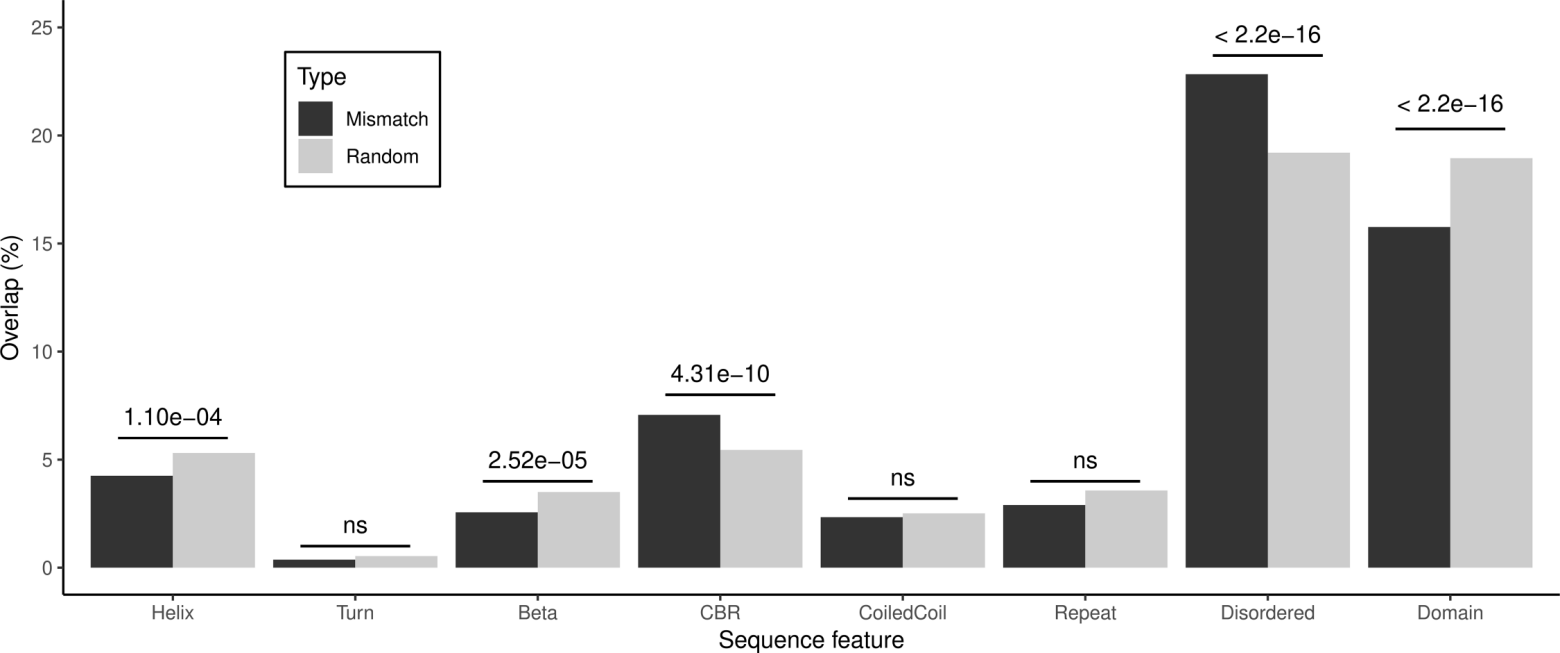
Overlap of human-specific residues (6210 mismatches) with sequence features annotated in human sequences (from UniProtKB), compared to the overlap of 100,000 random positions in the same human sequences. Fisher’s exact tests were performed to compare the sets; p-values are shown on top of the comparisons. CBR = Compositionally-biased regions.

We then studied the overlap of the human-specific substitutions with positional annotations provided by the UniProtKB database. These annotations may be overlapping in the database. These positions are significantly enriched in disordered regions, as well as in compositionally-biased regions (CBR) (Fig 2). On the other hand, they are significantly depleted in alpha helices, beta strands and domains in general.

Last, we performed enrichment analysis of the annotations of the proteins containing the set of human-specific residues with the highest conservation in the non-human primates (at least 10 out of the 11 studied non-human primates have the same amino acid). This was a subset of 1301 mismatches from 1095 different proteins (S3 File). We performed enrichment analyses using two different tools, DAVID v2025_1 [15] and PANTHER 19.0 [16]. In both cases, Fisher’s exact tests with Bonferroni corrections for multiple testing were used. The top annotation terms obtained in the DAVID tool were: protein binding (corrected p-value = 1.0e-5), composition bias: polar residues (corrected p-value = 6.5e-5), repeat: LRR 2 (corrected p-value = 4.8e-2), disordered (corrected p-value = 4.8e-2), and repeat: LRR 1 (corrected p-value = 4.8e-2). On the other hand, with PANTHER we found no enriched biological process, no enriched molecular function, and no enriched cellular component. Taken together, these results show that proteins containing human-specific residues are not particular in their functions: enriched terms confirm sequence features associated with non-globular structures.

## Discussion

Our species has many distinctive characteristics not shared with our closest relatives not only in cognitive and behavioural traits but also in anatomy, metabolism and physiology. These differences are coded in our genomes such that comparisons with closely related species are very helpful to understand their genetic basis. The first comparison of the human and chimpanzee genomes showed a very high degree of identity in the coding regions. We propose that if population variability is taken into account such differences will be further reduced, and if other primates are included, the number of human-specific variants will be much smaller. This study therefore was aimed to evaluate human-specific amino acid positions, accounting for variability within the human population, and comparing them across multiple primate species. By analyzing protein-coding sequences from 12 primates and incorporating data from the Genome Aggregation Database (gnomAD), we significantly reduced the number of human-specific amino acid differences from chimpanzees by 95%, when compared to the human-chimp substitutions obtained by comparing their reference proteomes. This reduction highlights that many of the previously identified differences are polymorphic within humans or present in other primates, so they are not human specific, and therefore very likely not relevant for the phenotype. This result, although not unexpected, is remarkable in the very low number of amino acid positions in the proteome that are specific to our species. We anticipate that once primate population diversity is considered (something we did not analyze in this study), human-specific positions will be further reduced. Now that the number of amino acid positions specific to humans has been significantly reduced, it will be very interesting to investigate which of them are relevant to the unique phenotypes displayed by our species.

Our analysis showed that the human-specific amino acid substitutions detected in this work are enriched in disordered and compositionally-biased regions, and consequently are depleted in structured domains and elements like helices and beta strands. Accordingly, recent results from our group have shown that low complexity regions, such as short tandem repeats [22] and poly amino acid tracks [23], are hotspots for mutation in the human genome. This is revealing mutation processes affecting protein regions that have regulatory roles and modulate protein interactions [24], while avoiding globular domains, which perform protein catalytic and structural functions. Our findings support the hypothesis that regulatory mutations, rather than changes that modify primary protein functions, account for significant biological differences between humans and other primates.

This research underscores the complexity of identifying truly species-specific genetic differences and the importance of considering population variability. It is very likely that the human-specific positions identified in this work will be further reduced once the variability of the other primate species are taken into account, since here we only analyzed the information in their reference genomes. Future studies focusing on the functional implications of these variations and their roles in regulatory processes will further our understanding of human uniqueness.

## Supporting information

S1 Fig Flowchart describing the steps followed to filter the initial mismatched positions between human and chimp one to one orthologs to obtain the set of human-specific residues. (PDF)

S2 Fig Distribution of the human-specific residues with respect to their relative position in the human proteins. In red, mismatches in the first and last decile of the human proteins.
(PDF)

S1 File List of 20,038 human-specific residues from 7052 different proteins, and the amino acid in the rest of the non-human primates. (XLS)

S2 File List of 6210 human-specific residues from 4475 different proteins, and the amino acid in the rest of the non-human primates. (XLS)

S3 File List of 1301 human-specific residues from 1095 different proteins, and the amino acid in the rest of the non-human primates. (XLS)
