# Supplementary figures and images for: Apparent differences between human and chimp proteomes are reduced when considering human population: Human specific variants are enriched in disordered and compositionally biased regions
Source: PLoS One. 2025 Jul 31;20(7):e0328504. doi: 10.1371/journal.pone.0328504 (PMC12312948; doi:10.1371/journal.pone.0328504)

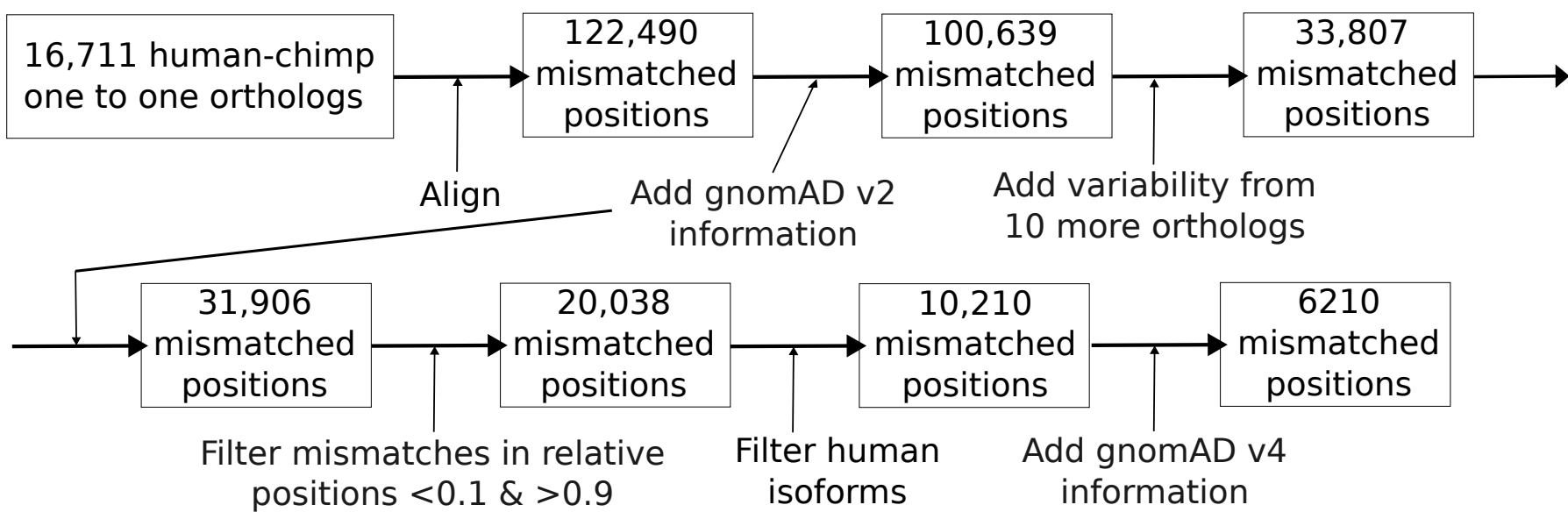

Supplement: S1 Fig — (PDF) [file pone.0328504.s001.pdf]

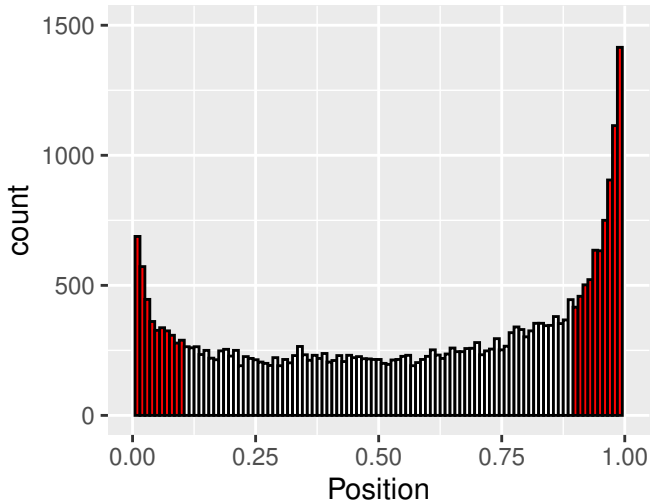

Supplement: S2 Fig — In red, mismatches in the first and last decile of the human proteins. (PDF) [file pone.0328504.s002.pdf]
